# Impact of 4 Weeks or More Immersive Virtual Reality on Quality of Life and Physical Activity in Older Adults: Systematic Review and Meta-Analysis

**DOI:** 10.2196/80820

**Published:** 2026-01-12

**Authors:** Iria Trillo-Charlín, Javier Bravo-Aparicio, Juan Avendaño-Coy, Héctor Beltrán-Alacreu

**Affiliations:** 1 Toledo Physiotherapy Research Group (GIFTO), Faculty of Physical Therapy and Nursing, University of Castilla-La Mancha, Toledo, Spain; 2 Toledo Physiotherapy Research Group (GIFTO), Castilla-La Mancha Health Research Institute (IDISCAM), Toledo, 45004, Spain; 3 Pain, Mental Health, Exercise and Technology Research Group (PAIN+MET), Faculty of Physical Therapy and Nursing, University of Castilla-La Mancha, Avenue de Carlos III, 45004 Toledo, Toledo, Spain, 34 967 599 200

**Keywords:** adverse events, immersive virtual reality, older adults, pain, perceived effort, physical activity, PRISMA, Preferred Reporting Items for Systematic Reviews and Meta-Analyses, quality of life

## Abstract

**Background:**

Population aging poses significant public health challenges. Older adults often face multimorbidity, functional decline, and diminished quality of life. While physical activity can mitigate these effects, adherence remains low. Immersive virtual reality (IVR) has emerged as a promising, engaging tool to promote physical and cognitive health in this population.

**Objective:**

The review aims to evaluate the effectiveness of IVR interventions lasting 4 weeks or more on quality of life, physical activity, pain, perceived effort, and adverse events in older adults.

**Methods:**

A systematic review and meta-analysis were conducted following PRISMA (Preferred Reporting Items for Systematic Reviews and Meta-Analyses) and Cochrane guidelines. Literature was searched across PubMed, Web of Science, PEDro, and Scopus, as well as sources of gray literature. Eligible studies included randomized controlled trials involving participants aged >60 years, using IVR via head-mounted display. Outcomes assessed included quality of life, physical activity, pain, perceived effort, and adverse events. Risk of bias and evidence certainty were assessed using Risk of Bias 2.0 and GRADE (Grades of Recommendation Assessment Development and Evaluation), respectively.

**Results:**

A total of 14 studies with 839 participants were included in the qualitative synthesis, of which 8 were eligible for quantitative meta-analysis. The pooled analysis showed a statistically significant moderate effect of IVR on quality of life (standardized mean difference [SMD]=0.48, 95% CI 0.1-0.8; *P*=.007), particularly in interventions lasting 10 to 12 weeks or involving more than 600 minutes of exposure. For physical activity, no significant differences were found between IVR and control groups (SMD=–0.2, 95% CI –0.7 to 0.4; *P*=.50). Evidence for secondary outcomes (pain, perceived exertion, and adverse events) was limited and largely qualitative, with inconsistent findings. Pain outcomes, assessed in 2 studies, indicated reductions in the IVR group, especially when multimodal approaches were used. Perceived effort was not systematically measured. Adverse events were generally mild, with cybersickness being the most reported issue.

**Conclusions:**

IVR interventions of 4 weeks or more appear to moderately improve quality of life in older adults, especially those with clinical vulnerabilities or in institutional settings. Although effects on physical activity were not significant, trends suggest potential with appropriate program design. Preliminary findings support IVR’s use in pain reduction, particularly when incorporating emotional and multisensory elements. The low incidence of adverse events suggests good tolerability. Overall, IVR is a promising and safe tool to support healthy aging, though further high-quality studies are needed to confirm these findings and assess long-term outcomes.

## Introduction

By 2030, the population of adults aged ≥65 years is expected to reach 994 million (12% worldwide), intensifying pressures on public-health systems [1]. From a biological perspective, aging is defined as a complex and progressive process that affects multiple systems, leading to a decline in functional capacity and increased vulnerability to various pathologies [2]. Aging is often accompanied by impairments such as loss of muscle strength, cognitive decline, and a heightened risk of falls and disability, all of which negatively impact the autonomy and quality of life of older adults [2-4].

In this context, it is crucial to identify effective strategies that support health and well-being in later life, with physical activity being one of the fundamental pillars for promoting healthy aging [5]. In older adults, physical activity is associated with better physical function, preservation of cognition, and enhanced quality of life. It is also linked to lower disability in activities of daily living, fewer falls, and a reduced risk of neurodegenerative diseases such as dementia [6-9]. Moreover, it has been linked to decreased mortality rates, and it is a leading modifiable determinant of healthy aging with a dose–response association to all-cause and cardiovascular mortality in older adults [10,11]. However, despite the available evidence, the proportion of older adults who meet physical activity recommendations remains suboptimal [11]. Several factors contribute to this, including lack of awareness of the benefits, fear of pain or falling, low motivation, and environmental barriers [12].

In this regard, immersive virtual reality (IVR) emerges as a potential nonpharmacological therapeutic alternative. Through 3D-simulated environments experienced via devices known as head-mounted displays, IVR offers an immersive experience in which users can interact with the virtual environment [13]. IVR’s sensorimotor immersion and playful elements may support engagement with physical activity, but current evidence is exploratory and based on a small feasibility trial [14]. Because IVR may enhance motivation and engagement, it is clinically and pragmatically important to test whether multiweek IVR programs can change physical activity behavior. Accordingly, our protocol designated physical activity as a coprimary outcome [15]. Additionally, its design allows for the adjustment of difficulty levels, making it a customizable alternative [16,17]—a feature considered essential in exercise prescription [18].

On the other hand, IVR has also proven useful in interventions aimed at cognitive stimulation and reminiscence, thereby expanding its range of applications in the context of active aging [19,20].

Currently, evidence suggests that IVR may benefit balance, mobility, cognition, and psychological well-being. However, some trials have reported mixed or modest effects [20-23]. These findings highlight the need for standardized outcome measures and adequately dosed programs in future research. These interventions have been implemented in both healthy individuals [21,24] and those with chronic medical conditions [22] or mild cognitive impairment [20]. However, existing reviews often involve short intervention periods [25]. This limits the ability to observe sustained long-term effects and may inflate immediate benefits [15,21]. Therefore, the focus is placed on programs delivered over periods that map onto the recall horizons of core quality-of-life instruments, a window that also coincides with the early emergence of neural and motor adaptations in response to training [26-30]. Trials in older adults have reported measurable gains over such intervals—cognition, balance, and functional outcomes [31-34]. Importantly, centering analyses on these exposure windows reduces the susceptibility of very brief interventions to novelty and Hawthorne effects [35]. In line with the World Health Organization’s (WHO) healthy aging agenda, which prioritizes functional capacity and well-being as central goals in aging societies, we selected quality of life and physical activity as primary outcomes. These measures are patient-centered indicators of healthy aging and modifiable behavior, making them particularly relevant assessment criteria [36].

Prior reviews in older adults have primarily established that virtual reality (VR) is acceptable and feasible, while noting that evidence for effectiveness remains limited [15]. Other syntheses have focused on exergames and outcomes such as balance in long-term care facilities, aggregating heterogeneous exposure durations and without a prespecified minimum dose [37]. More recent randomized controlled trial (RCT)-only reviews of head-mounted display VR suggest benefits for physical activity and broader well-being, but do not isolate ≥4-week programs as an a priori inclusion criterion [38]. To our knowledge, no systematic review has focused exclusively on IVR interventions in older adults with a minimum duration of ≥4 weeks as an a priori inclusion criterion.

The objective of this systematic review and meta-analysis is to evaluate the effectiveness of IVR interventions lasting 4 weeks or more on the quality of life, physical activity, pain, perceived effort, and adverse events in older adults.

## Methods

The protocol for this systematic review and meta-analysis was registered in the International Prospective Register of Systematic Reviews (CRD420251019170). It was conducted following the recommendations of the Cochrane Collaboration and the PRISMA (Preferred Reporting Items for Systematic Reviews and Meta-Analyses) guidelines (Checklist 1) [39]

### Search Strategy

A bibliographic search was completed between March 15, 2025, and June 15, 2025, in all the following databases: PubMed, Web of Science, PEDro, and Scopus. Additionally, we systematically searched for gray literature. This included searches in ClinicalTrials.gov, the WHO International Clinical Trials Registry Platform, ProQuest Dissertations & Theses Global, and medRxiv. Search strategies used are available in Multimedia Appendix 1 [14,18,24,29,40-53].

We also carried out a “snowball” search to identify additional studies by searching the reference lists of publications eligible for full-text review.

### Inclusion and Exclusion Criteria

Inclusion criteria were defined according to the PICOS (population, intervention, comparison, outcome, study design) framework: (1) we included studies only if all participants were aged ≥60 years at baseline; (2) IVR intervention, alone or combined with other therapies, lasting 4 weeks or more; (3) the comparison group can be control, placebo, or another type of intervention; (4) outcome measures related to physical activity, quality of life, pain intensity, perceived effort, and adverse events; and (5) randomized controlled trials (RCTs), crossover clinical trials, and randomized mixed methods studies.

Exclusion criteria were: (1) studies with participants aged under 60 years, although the average age is 60 years or older; and (2) studies whose intervention is defined as immersive but does not use the head-mounted display.

No language restrictions were applied while searching.

### Study Selection

Once the research question was defined, studies were identified and screened accordingly. Following the search strategy, all references were imported into Rayyan (Rayyan Systems Inc) to exclude duplicate studies [54].

Two independent researchers (IT-C and JB-A) conducted the study selection based on the predefined inclusion and exclusion criteria. In cases of disagreement, a third researcher (HB-A) was consulted to resolve discrepancies and reach a consensus.

### Data Extraction

First, two researchers (IT-C and JB-A) independently extracted key information from the included studies. In case of discrepancies, a third researcher was consulted to resolve disagreements. The extracted data included: first author and year of publication, country, study design, sample size, age, diagnosis, setting, type of intervention of the groups, hardware and software of VR used, time of intervention (total amount of time in h, session time, frequency, and number of wk), outcomes, and follow-up period.

In the second phase, quantitative data for both primary and secondary outcomes were extracted. For the primary outcomes—physical activity and quality of life—as well as for the secondary outcomes of pain, perceived effort, and adverse events, mean and SD values were collected. When studies reported both change scores and final values, the final values were prioritized for analysis. When information regarding any of the above was unclear, we contacted authors of the reports to provide further details.

If data were only available in graphs, the graph digitization software GraphGrabber 2.0.2 (Quintessa Ltd) was used for extraction [55].

### Risk of Bias

The methodological quality of the included studies was independently assessed by 2 researchers using the Cochrane Risk of Bias 2.0 tool for both parallel and crossover designs [56]. In instances where discrepancies arose between the two primary researchers, a third independent researcher was consulted to resolve disagreements. This tool evaluates the risk of bias across 5 domains: randomization process, missing outcome data, measurement of outcomes, selection of reported results, and deviations from intended interventions.

In addition, selective reporting will be judged by comparing published outcomes with registered protocols when available.

### Main Outcomes

The primary outcomes of this study were quality of life and physical activity. Any objective or self-reported measure of these outcomes was considered eligible for inclusion, provided the instruments used demonstrated evidence of validity and reliability. No restrictions were placed on the number or timing of assessment time points (eg, baseline, postintervention, and follow-up).

The secondary outcomes included pain intensity, perceived effort, and adverse events. The same eligibility and selection criteria were applied, with preference given to the numeric rating scale (NRS) and visual analog scale (VAS) for pain intensity [57].

### Statistical Analysis

A meta-analysis was conducted to estimate the overall effect of IVR interventions on quality of life and physical activity levels in older adults. As the included studies used different scales to assess these outcomes, the standardized mean difference (SMD) was used as the effect size measure. In all cases, higher scores indicated better outcomes, except for the study by Rodríguez-Fuentes et al [43], which used the Parkinson Disease Questionnaire (PDQ-39) scale, where lower scores reflect a better quality of life.

All hypothesis tests were 2-tailed, with statistical significance set at *α*=.05. Effect estimates are reported with 95% CIs. The inverse variance method was applied using a random-effects model, considering the expected clinical and methodological heterogeneity among studies. For each outcome, 95% CI were calculated, and a significance level of *P*<.05 was established. The magnitude of the effect was interpreted according to the Cohen criteria: small effect (SMD approximately 0.2), moderate (approximately 0.5), and large (≥0.8).

Statistical heterogeneity was assessed using the chi-square test, the 𝜏² estimate, and the *I*² statistic. Heterogeneity was established as low for *I*^2^=25%, moderate for *I*^2^=50%, and high for *I*^2^=75%. Subgroup analyses were conducted based on type of control group (active vs passive), intervention duration in weeks (6‐8wk vs 10‐12 wk), and total exposure time (180‐480 min vs 600‐1800 min). In addition, a sensitivity analysis was conducted by sequentially excluding each individual study to assess its impact on the overall effect size and heterogeneity.

All statistical analyses were performed using Review Manager (RevMan) version 5.4.1. The certainty of the evidence will be assessed using the GRADE (Grades of Recommendation Assessment Development and Evaluation) approach [58].

### Deviations From Protocol

Several deviations from the International Prospective Register of Systematic Reviews protocol occurred. The mental component of the 12-item Short Form Survey (SF-12) was not analyzed to avoid conceptual overlap (Multimedia Appendix 1 [14,18,24,29,40-53]). Publication bias assessment was planned but not performed due to <10 studies per outcome (Multimedia Appendix 1 [14,18,24,29,40-53]). When required, means and SDs were estimated from median (IQR) following validated procedures (Multimedia Appendix 1 [14,18,24,29,40-53]). Subgroup analyses were restricted to comparator type, intervention duration, and total exposure; definitions and limitations are detailed in Multimedia Appendix 1 [14,18,24,29,40-53].

## Results

### Study Selection

**Figure 1.**
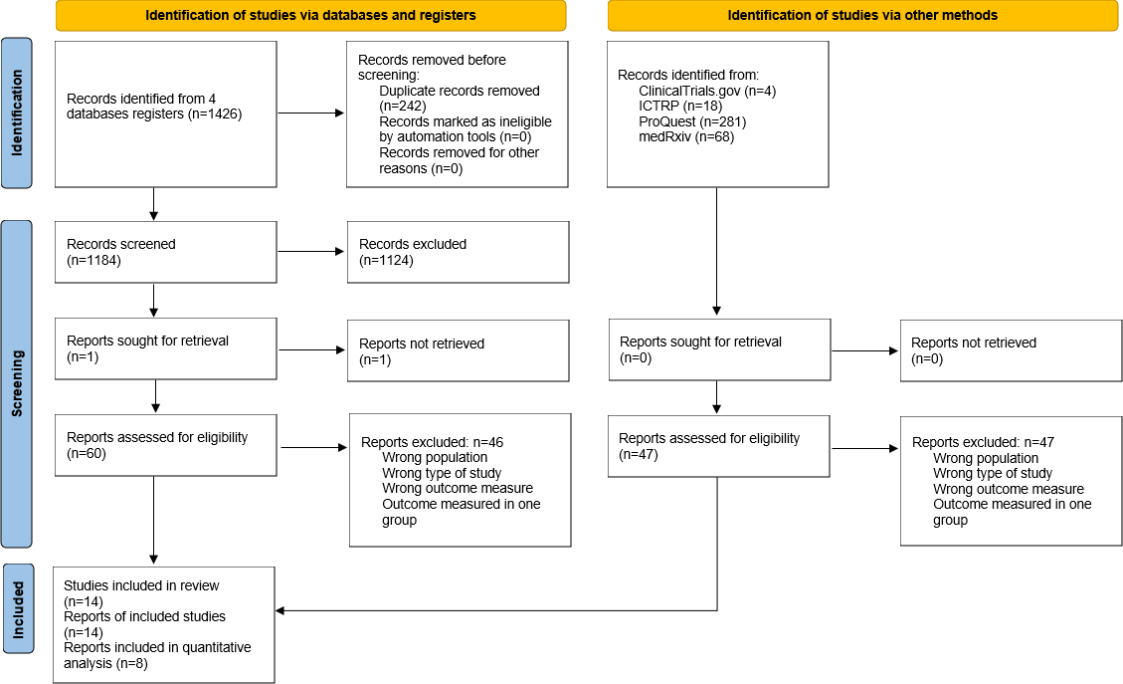
Flow diagram in this review. ICTRP: International Clinical Trials Registry Platform.

The selection process is illustrated in the PRISMA 2020 flow diagram (Figure 1) in this review.

We found 1426 records in database searching. After duplicate removal, we screened 1184 records, from which we reviewed 60 full-text documents. Later, we searched documents that cited any of the initially included studies as well as the references of the initially included studies. However, no extra studies that fulfilled the inclusion criteria were found in these searches.

Ultimately, 14 trials met the inclusion criteria and were included in the systematic review. Of these, 8 studies were included in the meta-analysis. The remaining 6 studies were excluded from the quantitative synthesis because they did not report outcome measures corresponding to the primary endpoints analyzed in the meta-analysis (ie, quality of life or physical activity).

### Study Characteristics

This systematic review included 14 RCTs published between 2021 and 2025 [14,19,25,43-53], with a total of 839 older adults and sample sizes ranging from 9 to 293 participants. While most studies included older adults without specific diagnoses [14,19,25,44,45], others focused on populations with cognitive impairment or frailty [46-48,50], balance and mobility issues [51], Parkinson disease [43], knee osteoarthritis [53], chronic low back pain [52], or individuals with joint arthroplasty [49].

Regarding settings, 10 studies were conducted in community centers or nursing homes [19,43-48,50-52], 3 in laboratory conditions [44,52], and 2 at home [14,53].

Most studies focused on physical activity delivered via VR, using environments that required movement [14,44,51]. In some cases, VR-based physical activity was combined with usual care or conventional rehabilitation, such as occupational therapy or kinesiotherapy [45,49]. Other interventions emphasized cognitive training delivered via VR [46], sometimes including underdesk ergometers for simultaneous stimulation [47,48]. Some protocols included education sessions, followed by structured VR-based exercise programs [25,53].

A few studies explored more therapeutic applications of VR, such as immersive reminiscence therapy [19], or multimodal pain management programs that integrated psychoeducation and movement therapy [52]. One study used VR to simulate daily living environments, aiming to promote autonomy in participants with cognitive frailty [50].

Control conditions varied: some used usual care [19,45,47,50], others applied active comparators such as conventional rehabilitation or group-based exercise [14,43,48,49,51-53] and a few used no-intervention or educational controls [25,44,46].

Session durations typically ranged from 15 to 60 minutes, with frequencies varying from 1 to 5 times per week over periods from 4 to 12 weeks. The longest interventions, by Lo et al [53] and Rodríguez-Fuentes et al [43], lasted 12 weeks.

Detailed tables summarizing the characteristics of each included study are provided in Table 1 (study characteristics) and Table 2 (intervention characteristics). In addition, detailed specifications of software, hardware, and outcome instruments are provided in Multimedia Appendix 1 [14,18,24,29,40-53].

**Table 1.** Study characteristics.

Study ID	Study design	Sample size	Age (y)	Diagnosis	Setting
Barsasella et al (2021) [44]	RCT^a^	n=60; VRG^b^ n=29; CG^c^ n=31	NI^d^	NSD^e^	Laboratory-based
Campo-Prieto et al (2022) [45]	RCT	n=24; VRG n=13; CG n=11	VRG: mean 85.08 (SD 8.48) CG: mean 84.82 (SD 8.1)	NSD	Clinical-based
Chiu et al (2023) [46]	RCT	n=60; VRG n=30; CG n=30	VRG: mean 80.7 (SD 8.8) CG: mean 80 (SD 7.9)	Cognitive impairment	Clinical-based
Drazich et al (2023) [24]	Pilot RCT	n=20; VRG n=10; CG n=10	Mean 74.1 (SD 6.5)	NSD	Clinical-based
Kershner et al (2024) [14]	Pilot RCT	n=9; VRG n=5; CG n=4	Mean 66.8 (SD 4.8)	NSD	Home-based
Khirallah Abd el Fatah et al (2024) [18]	RCT	n=60. VRG n=20; RT group n=20; CG n=20	Mean 66.68 (SD 4.22)	NSD	Clinical-based
Kwan et al (2021) [48]	Pilot RCT	n=15; VRG n=9; CG n=8	Median: 74 (IQR 9,5)	Cognitive frailty	Clinical-based
Kwan et al (2024) [47]	RCT	n=293; VRG n=146; CG n=147	Mean 74.5 (SD 6.8)	Cognitive frailty	Clinical-based
Lo et al (2024) [53]	Mixed methods pilot RCT	n=30; VRG n=15; CG n=15	Median: 63.5 (IQR 61.8‐66.3)	Knee osteoarthritis	Home-based
Mazurek et al (2023) [49]	RCT	n=68; VRG n=34; CG n=34	Mean 69.59 (SD 6.16)	Hip or knee joint arthroplasty surgeries	Laboratory-based
Rodríguez-Fuentes et al (2024) [43]	RCT	n=52; VRG n=30; CG n=22	Mean 70.79 (SD 6.59)	Parkinson	Parkinson Association
Sekar et al (2024) [51]	RCT	n=60; VRG=30; CG=30	NI	Balance and mobility issues	Clinical-based
Stamm et al (2022) [52]	Pilot RCT	n=22; VRG n=11; CG n=11	VRG: mean 75.0 (SD 5.8) CG: mean 75.5 (SD 4.39)	Chronic low back pain	Laboratory-based
Zheng et al (2025) [50]	RCT	n=66; VRG n=33; CG n=33	Mean 80.20 (SD 9.14)	Cognitive frailty	Clinical-based

a RCT: randomized controlled trial.

b VRG: virtual reality group.

c CG: control group.

d NI: no information.

e NSD: no specific diagnosis.

**Table 2.** Intervention characteristics.

Study ID	Experimental group (s)	Control group	Frequency	Follow-up
Barsasella et al (2021) [44]	PA^a^ via VR^b^	No intervention	15’ 2 t/w^c^ 6 weeks TT:^d^ 180’	—^e^
Campo-Prieto et al (2022) [45]	Usual care+PA via VR	Usual care (occupational therapy and memory workshops)	6’ 3 t/w 10 weeks TT: 180’	—
Chiu et al (2023) [46]	Cognitive training intervention via VR	No intervention	60’ 1 t/w 8 weeks TT: 480’	—
Drazich et al (2023) [24]	PA education+VR	1 session of PA education	40’ 2 t/w 8 weeks TT: 640’	—
Kershner et al (2024) [14]	PA via VR	PA via group videoconference	45‐60’ minimum/w 4 weeks TT: 180‐240’ minimum	—
Khirallah Abd el Fatah et al (2024) [18]	EXP 1: IVR^f^ reminiscence therapy EXP 2: Traditional reminiscence therapy	Usual care (daily personal care, primary nursing care, medical care)	30‐45’ 2 t/w 6 weeks TT: 360‐540’	3 months
Kwan et al (2021) [48]	Motor and cognitive training on VR+underdesk ergometer	Cognitive training on tablet computers and motor training cycling on ergometer	30’ 2 t/w 8 weeks TT: 480’	—
Kwan et al (2024) [47]	Motor and cognitive training on VR+underdesk ergometer	Usual care (activities provided by the community centers)	20‐30’ 2 t/w 8 weeks TT: 320‐480’	—
Lo et al (2024) [53]	Health talk+lower limb exercises via VR	Lower limb exercises	30’ 5 t/w 12 weeks TT: 1800’	—
Mazurek et al (2023) [49]	Relaxing VR+conventional rehabilitation	Conventional rehabilitation (kinesiotherapy, ergotherapy, laser therapy/magnetic therapy/electrotherapy)	VR sessions: 20’ 2 t/w 4 weeks TT: 160’	—
Rodríguez-Fuentes et al (2024) [43]	Cycloergometer+VR	Static Cycling using Smart Cycloergometers	25’ 2 t/w 12 weeks TT: 600’	—
Sekar et al (2024) [51]	Balance and mobility exercises with VR	Balance and mobility exercises	2 t/w 8 weeks	—
Stamm et al (2022) [52]	Movement therapy and psychoeducation via VR	Conventional multimodal pain therapy (chair-based group exercises and psychoeducation units)	30’ 3 t/w 4 weeks TT: 360’	—
Zheng et al (2025) [50]	Scenarios with daily environments via VR	Usual care (nursing care, and routine activities like finger exercises and holiday paper cutting)	45’ 2 t/w 12 weeks TT: 1080’	—

a PA: physical activity.

b VR: virtual reality.

c t/w: times/week.

d TT: total time.

e —: not available.

f IVR: immersive virtual reality.

### Main Outcomes

#### Quality of Life

Among all 6 studies assessed quality of life using various validated tools such as EuroQol VAS [44,53], SF-12 [45,50], World Health Organization Quality of Life Scale Brief Version (WHOQOL-BREF) [43,46] and PDQ-39 [46,50].

Overall, 5 studies reported improvements in quality of life following IVR interventions. Chiu et al [46] and Zheng et al [50] found the most notable effects, with WHOQOL-BREF scores nearly doubling and significant gains in SF-12 mental health, respectively. Only Barsasella et al [44] found no overall differences in EuroQol 5D-3L scores between groups, though improvements were noted in specific domains such as pain and anxiety.

#### Physical Activity

Among all 3 studies assessed physical activity using both self-report and objective measures (eg, Yale Physical Activity Survey, Garmin Vivosmart 4, and ActivPAL accelerometry) [14,25,53].

Drazich et al [24] observed modest gains in vigorous activity within the VR group, despite stable weekly activity levels. Kershner et al [14] reported greater gains in steps and vigorous activity for the video conference group, though the VR group improved more in functional capacity. Lo et al [53] found slightly higher metabolic equivalent of tasks in the VR group, but without significant differences.

#### Intensity of Pain

Out of all 2 studies addressed pain. Lo et al [53] used the NRS and Western Ontario and McMaster Universities Osteoarthritis Index subscale in patients with osteoarthritis, showing reductions in both scores within the VR group (NRS: 5.93-4.78; Western Ontario and McMaster Universities Osteoarthritis Index: 189.5-160.2), with minimal changes in controls. However, these differences did not reach statistical significance when comparing IVR to control, suggesting no conclusive effect attributable to the intervention. Mazurek et al [49] reported a significant drop in VAS scores in the VR group (5.27-0.88), exceeding improvements in the control group.

#### Perceived Effort

None of the included studies systematically assessed perceived effort using validated tools such as the Borg rating of perceived exertion scale or comparable measures.

#### Adverse Events

Adverse events were reported in 7 of the included studies, focusing on cybersickness [14,19,25,45,47,48,53]. Overall, most studies reported minimal or mild adverse events. In the pilot study of Kwan et al [47,48], 1 participant in the VR group withdrew early due to persistent symptoms of cybersickness, while the RCT reported low incidence rates (0.7%-3%) across 293 participants. Kershner et al [14] observed mild symptoms present in some participants. Lo et al [53] found that 5 out of 15 participants in the VR group reported mild adverse events such as dizziness or visual fatigue, though these did not result in discontinuation.

### Risk of Bias in Studies

Most studies were judged to be at high risk of bias, except for 3 studies that showed a moderate risk [49,50,53]. All studies demonstrated low risk in domain 3 (missing outcome data). In domain 4 (measurement of the outcome), all but 3 studies were rated as high risk [49,50,53]. In domain 5 (selection of the reported result), 3 studies were rated as low risk, while the remaining studies were judged to have unclear risk.

**Figure 2.**
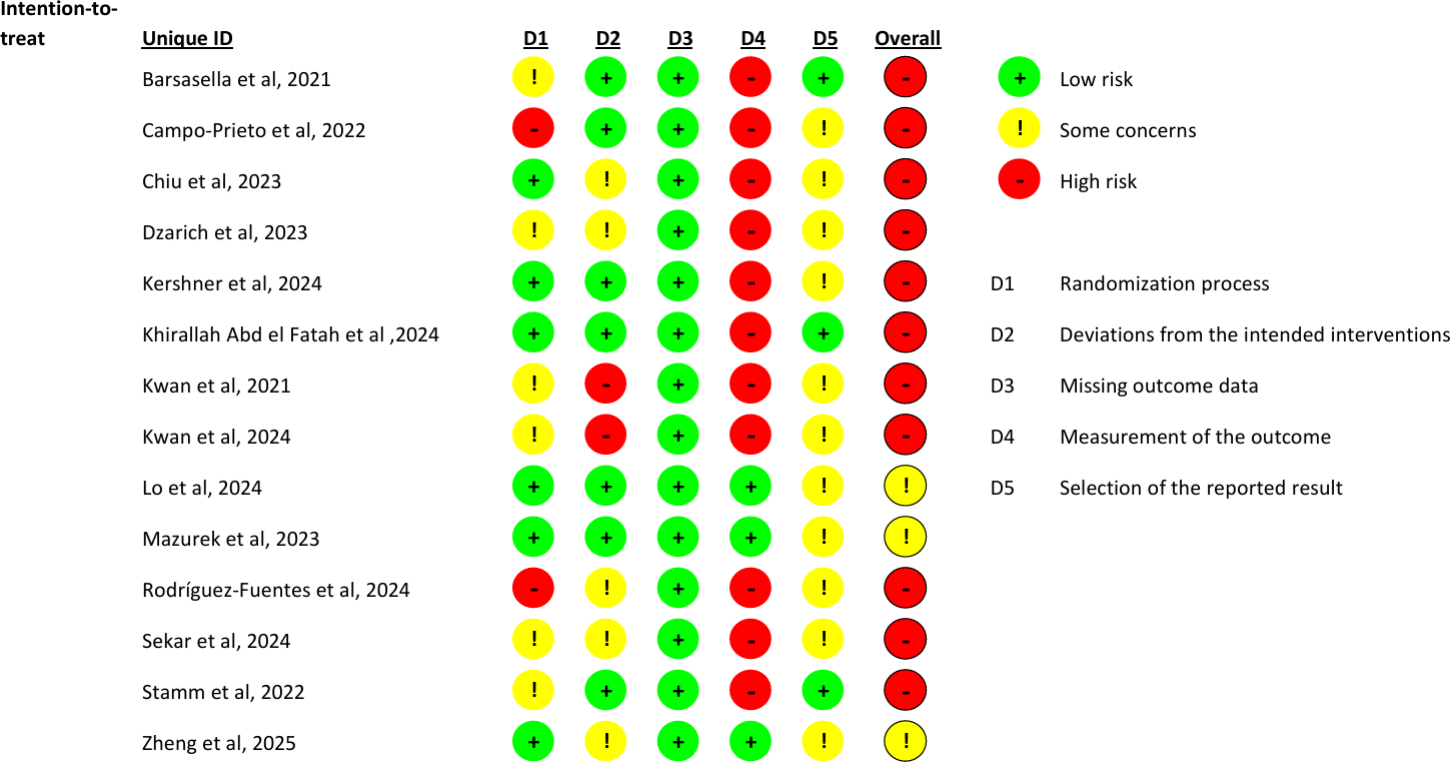
Risk of bias [14,18,24,43-53].

As shown in Figure 2, the agreement rate achieved between the 2 researchers who completed risk of bias assessment was 81.43%. In case of disagreement, a third researcher resolved it.

### Results of Syntheses

#### Quality of Life

**Figure 3.**
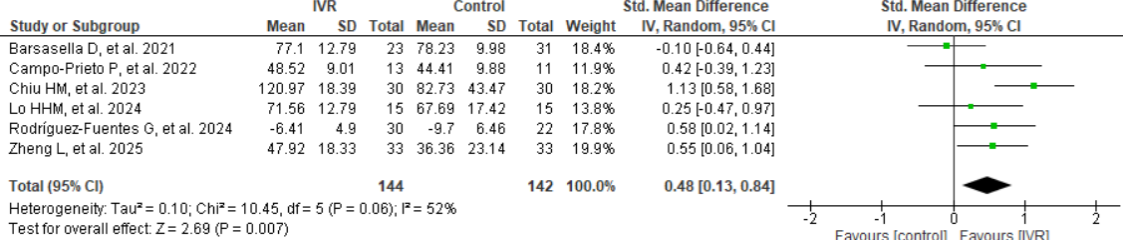
Effect of immersive virtual reality (IVR) on quality of life [43-46,50,53].

As shown in Figure 3 (effect of IVR on quality of life), a total of 6 RCTs including 286 participants (IVR group: n=144; control group: n=142) were synthesized to examine the effect of IVR interventions on quality of life in older adults. The meta-analysis yielded a statistically significant moderate effect in favor of IVR compared to control conditions (SMD=0.48, 95% CI 0.1-0.8; *P*=.007). Heterogeneity was moderate (*I*²=52%; *P*=.06; 𝜏²=0.10), indicating some variability across studies.

As shown in Table 3 subgroup analyses were performed to explore the potential influence of comparator type, intervention duration in weeks, and total intervention time in minutes.

**Table 3.** Subgroup analysis.

Subgroup	Studies, n	Participants, n	Random-effects model		Heterogeneity *I*^2b^ (%)	Between subgroups difference	
			SMD^a^ (95% CI)	*P* value		Chi-square (*df*)	*P* value
Comparator type						0.02 (1)	.89
Passive control	4	110	0.50 (−0.04 to 1.04)	.07	71		
Active control	2	82	0.48 (0.12 to 0.84)	.05	0		
Weeks of intervention						0.00 (1)	.97
6‐8	2	120	0.51 (−0.69 to 1.72)	.40	90		
10‐12	4	172	0.48 (0.18 to 0.79)	.002	0		
Minutes of intervention						0.00 (1)	.99
180‐480	3	138	0.49 (−0.31 to 1.28)	.23	80		
600‐1800	3	286	0.49 (0.13 to 0.84)	.003	0		

a SMD: standardized mean difference.

b *I*2: inconsistency index.

Within-subgroup analyses showed statistically significant effects in some conditions. For instance, a significant effect was observed in the subgroup receiving 600‐1800 minutes of intervention (SMD=0.49, 95% CI 0.13-0.84; *P*=.003), in contrast to the nonsignificant effect in the 180‐480 minutes group. Similarly, the 10‐12 week intervention subgroup showed a statistically significant moderate effect (SMD=0.48, 95% CI 0.18-0.79; *P*=.002), while the 6‐8 week group did not (*P*=.40).

Regarding comparator type, significant effects were observed in the active control group (*P*=.05), but not in the passive control group (*P*=.07). However, despite these within-subgroup differences, the overall between-subgroup comparisons were not statistically significant for any of the 3 variables assessed: comparator type (*P*=.89), duration in weeks (*P*=.97), or total minutes of intervention (*P*=.99). These findings suggest that while effect sizes may vary descriptively across subgroups, such differences are not supported statistically.

**Figure 4.**
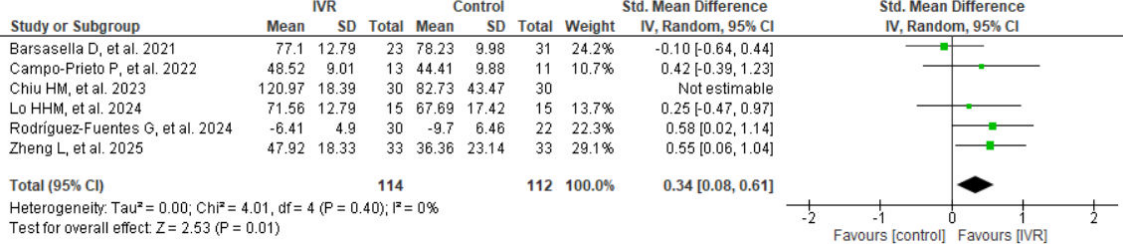
Sensitivity analysis of IVR effects on quality of life. IVR: immersive virtual reality [43-46,50,53].

As shown in Figure 4 (sensitivity analysis of IVR effects on quality of life), a sensitivity analysis was conducted excluding the study by Chiu et al [46], which presented the largest effect size (SMD=1.1). When this study was removed, the overall effect in favor of IVR remained statistically significant (SMD=0.3, 95% CI 0.08-0.6; *P*=.01), although the magnitude of the effect was reduced. Notably, heterogeneity was eliminated (*I*²=0%; 𝜏²=0.00), suggesting that this study contributed substantially to the observed heterogeneity in the main analysis.

#### Physical Activity

Among all 3 RCTs involving a total of 59 participants (IVR group: n=30; control group: n=29), IVR was assessed on physical activity in older adults. The meta-analysis revealed no statistically significant difference between groups (SMD=−0.2, 95% CI −0.7 to 0.4; *P*=.50).

**Figure 5.**
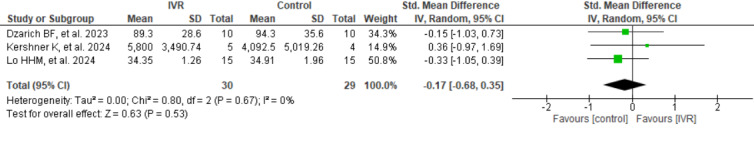
Effect of immersive virtual reality (IVR) on physical activity [14,24,53].

As shown in Figure 5 (effect of IVR on physical activity), heterogeneity among studies was minimal (*I*²=0%; *P*=.70; 𝜏²=0.00), indicating high consistency in the direction and magnitude of effects across studies. Due to the small number of studies, no subgroup or sensitivity analyses were performed for this outcome.

### Certainty of Evidence

**Figure 6.**
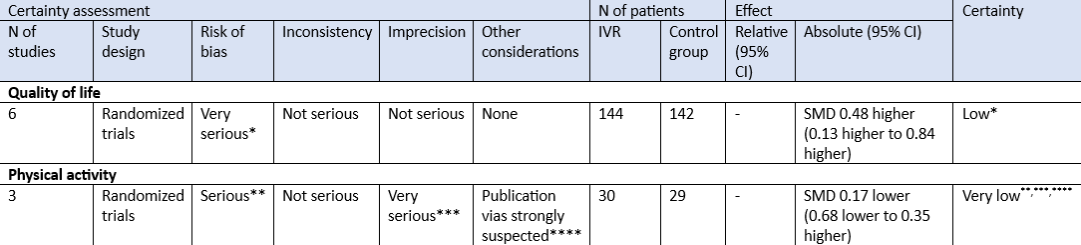
Assessment of evidence according to GRADE (Grades of Recommendation Assessment Development and Evaluation). IVR: immersive virtual reality; SMD: standardized mean difference. *Out of 6 studies, 4 contributing to this outcome were judged to have high risk of bias, particularly due to issues in the randomization process, deviations from intended interventions, and selective reporting. The remaining 2 had some concerns. **Out of the 3 studies, 2 were judged to have high risk of bias, particularly in randomization and outcome assessment. The overall certainty was downgraded due to methodological limitations. ***The CI was wide and included both meaningful benefit and harm. The small sample size and lack of statistical significance led to downgrading by 2 levels. ****Only 3 studies were included in the meta-analysis for physical activity, which prevents formal assessment of publication bias. Due to the small number of trials and likelihood of selective reporting, strong suspicion of publication bias was considered.

As shown in Figure 6 (assessment of evidence according to GRADE), the certainty of the evidence for the outcome quality of life was rated as low. Downgrading was applied due to very serious concerns related to the risk of bias. For the outcome of physical activity, the certainty of the evidence was rated as very low. Downgrading was applied due to serious risk of bias, very serious imprecision, and strong suspicion of publication bias.

## Discussion

### Principal Findings

To our knowledge, this review is the first to focus exclusively on IVR interventions in older adults with a minimum duration of ≥4 weeks as a predefined inclusion criterion. The findings reveal a statistically significant moderate effect of IVR on quality of life, especially in individuals with clinical vulnerability or living in institutional settings. In contrast, no significant effects were observed for physical activity, and the direction of the results across included studies was inconsistent. Although some isolated findings indicated minor improvements in specific physical activity parameters, these were not replicated across trials and were derived from interventions of limited intensity or duration. As such, current evidence does not support definitive conclusions regarding the impact of IVR on physical activity, though it highlights important considerations for future program design. On the other hand, given the limited number of studies and heterogeneous measurement, secondary outcomes (pain, perceived exertion, and adverse events) could not be robustly quantified. We retained these outcomes because they were prespecified in our protocol and are clinically salient, but current evidence is insufficient to support firm conclusions. Additionally, preliminary results indicate potential benefits for pain reduction, and IVR was well tolerated, with low incidence of mild adverse events.

### Quality of Life

Our findings suggest a moderate, statistically significant improvement in quality of life with IVR versus control; however, the certainty of evidence is low due to risk of bias. The observed effect size—approaching the 0.5 threshold commonly regarded as clinically meaningful in geriatric interventions—suggests a relevant improvement in this population. The psychological sense of presence elicited by IVR may diminish the perception of aversive stimuli such as pain or anxiety and promote emotional regulation, intrinsic motivation, and well-being [59,60].

Notably, the most pronounced benefits were evident among older adults with clinical diagnoses, indicating efficacy in individuals with functional vulnerabilities. This observation is consistent with previous research reporting cognitive gains in populations with mild cognitive impairment [61]. Moreover, interventions implemented in residential or institutional environments tended to yield more consistent and favorable outcomes. In support of this, Li et al [62] reported that IVR experiences in nursing homes enhanced not only well-being and social engagement but were also perceived as meaningful and motivating by participants.

Regarding intervention characteristics, programs incorporating cognitive or functional components generated more substantial improvements than those centered exclusively on physical exercise. This may reflect the inherently multidimensional nature of quality of life, which integrates cognitive, emotional, and social dimensions in addition to physical health [63].

In terms of duration, longer interventions (10‐12 wk or exceeding 600 min) could be associated with more reliable improvements in quality of life. This finding is aligned with results from Vasodi et al [64], who reported that extended IVR programs led to better outcomes in older adults’ mood and well-being, potentially due to increased engagement, gradual adaptation, and the consolidation of behavioral changes over time.

### Physical Activity

The effectiveness of IVR in promoting physical activity among older adults appears to be highly contingent on intervention design; however, in our review, the evidence is very uncertain. The meta-analysis included only 3 randomized trials and showed no statistically significant differences between IVR and control groups, with inconsistent directions of effect across studies. Coupled with the very low certainty of evidence, small total sample size, and wide confidence intervals, these findings should be interpreted as hypothesis-generating rather than decision-informing. These findings align with previous evidence suggesting that brief, lab-based programs are generally insufficient to generate sustained behavioral change. For instance, studies by Lo et al [53] and Drazich et al [24] reported only modest or transient increases in activity, likely due to seated, low-intensity exercises and short durations. In contrast, research in younger adults shows IVR can elicit greater physiological responses—such as increased oxygen consumption and enjoyment—when compared to traditional 2D formats, likely due to enhanced emotional engagement [65].

The success of longer, home-based interventions like those reported by Dinet and Nouchi [66] may reflect the critical importance of habit formation and environmental integration, which require extended exposure periods and real-world application contexts. However, such outcomes seem contingent on the intervention’s ability to integrate into daily routines, adapt to user capacity, and sustain motivation over time.

A further limitation is the reliance on self-reported physical activity measures, which are subject to bias. Future research should use wearable devices with validated protocols for objective monitoring. Overall, IVR can support physical activity, but only if programs are engaging, adaptable, and promote long-term autonomous use.

### Intensity of Pain

The effects of IVR on pain intensity in older adults show heterogeneous results, probably conditioned by the design of the interventions and the characteristics of the participants. In the pilot study by Lo et al [53] with older people with knee osteoarthritis, the differences in pain intensity did not reach statistical significance compared to the control group, which may be attributed to the limited format of the intervention, focusing exclusively on strength without aerobic elements or relevant visual distracters.

In contrast, the study by Mazurek et al [49] shows more robust results: after 8 sessions of IVR with psychotherapeutic approach and immersive relaxation, a significant reduction in pain was observed, with a significant difference compared to the control group. This effect could be explained by the inclusion of psychological components, such as attentional distraction, emotional reinforcement, and the use of therapeutic metaphors, which have been associated with a downward modulation of pain in previous neurobiological studies [67,68].

Additional studies reinforce this approach. Li et al [69] observed a significant reduction in chronic low back pain in older adults after 8 weeks of IVR combined with functional exercise, highlighting increased adherence and reduced analgesic use in the IVR group. Taken together, the evidence suggests that IVR may be effective in reducing pain in older adults, especially when applied with a multisensory, emotional, and adaptive approach.

### Adverse Events

The results of this review suggest that IVR is generally well tolerated by older adults, with a low incidence of adverse events, mainly related to mild symptoms of cybersickness (dizziness, nausea, and visual fatigue). Studies such as those by Campo-Prieto et al [45], Drazich et al [24], or Khirallah et al [18] reported no adverse events, while others, such as Lo et al [53], did observe mild symptoms in one third of participants, with no related dropouts. In Kwan et al [47,48], one withdrawal due to persistent cybersickness was reported, but in their later study, with a larger sample size, the incidence was low (0.7%‐3%). These findings are consistent with previous reviews. Weech et al [70], in a systematic review, identified that symptoms of cybersickness are common in immersive environments, but their severity tends to be mild and dependent on factors such as content type, duration of exposure, and individual characteristics. Stanney et al [71] emphasized the importance of individualized visor fit, especially interpupillary distance, noting that poor fit significantly increases discomfort, especially in women.

Overall, the evidence suggests good overall tolerability, although not without some episodes.

### Clinical Implications

The findings suggest that IVR may be a valuable clinical tool to enhance the quality of life in older adults, particularly those with functional limitations or in institutional settings. Given the predominance of high risk of bias across several trials and low to very low certainty by GRADE, the pooled effects should be interpreted as signals of possible benefit, not as precise estimates for clinical decision-making.

Interventions combining cognitive, physical, and motivational elements show greater effectiveness. Although physical activity outcomes were not significant, appropriate program design appears crucial for adherence. Preliminary evidence also supports IVR’s use in pain management, especially with emotionally engaging approaches. Its low rate of adverse events indicates good tolerability. With proper device adjustment and supervision, IVR can be progressively integrated into geriatric rehabilitation across various care environments.

From a policy perspective, our findings align with the UN Decade of Healthy Aging, particularly its priority on maintaining functional ability and person-centered care. IVR could act as an engagement-enhancing tool when embedded within routine health and social care services [72]. Operationally, its integration should be guided by WHO’s integrated care for older people pathways in primary and community care and adhere to the principles of the Global Strategy on Digital Health—namely interoperability, equity, and evidence-based implementation [72].

### Limitations

This systematic review and meta-analysis present several limitations related to its design and execution. First, although the search strategy was comprehensive and included 4 major databases, relevant studies indexed in other sources may have been missed. However, no language restrictions were applied, which mitigates selection bias. Second, the number of studies eligible for inclusion in the meta-analysis was limited, particularly for some outcomes such as physical activity and pain, reducing the statistical power and precision of the effect estimates. Additionally, due to the low number of included trials per outcome (n<10), we could not perform funnel plots or the Egger test to formally assess publication bias. Third, the heterogeneity in outcome measurement instruments and reporting formats across studies made it necessary to apply specific criteria for data inclusion, such as prioritizing final values over change scores and estimating means and SD when only medians and interquartile ranges were reported. These decisions, although methodologically justified, may introduce some degree of imprecision. Finally, some planned subgroup analyses could not be conducted due to insufficient data availability. While sensitivity analyses were performed where possible, the overall ability to explore sources of heterogeneity was limited.

A wide range of instruments was used to assess quality of life and physical activity (SF-12, EuroQol-5 Dimensions, WHOQOL-BREF, PDQ-39, accelerometers, and self-report surveys). While we synthesized conceptually similar constructs, instrument heterogeneity reduces scale-specific interpretability and likely contributed to between-study variance.

Future RCTs should address current evidence gaps by prioritizing: (1) the use of standardized and psychometrically validated outcome measures, particularly for physical activity and perceived effort; (2) objective monitoring of physical activity levels through wearable devices to reduce reliance on self-report; (3) long-term follow-up assessments to determine the durability of IVR effects on quality of life and functional outcomes; and (4) cost-effectiveness analyses to evaluate the feasibility of implementing IVR programs in real-world geriatric care settings; and (5) future studies should focus on the analysis of contextual factors—such as delivery model (standalone IVR vs adjunct), supervision intensity, and care setting—as potential effect modifiers. In addition, the main bias in the review was due to the unblinding of assessors and nonregistered protocols; therefore, future studies should (1) blind outcome assessors, and (2) prospectively register protocols with a prespecified analysis plan to prevent selective reporting.

### Conclusions

IVR interventions lasting 4 weeks or more appear to moderately improve quality of life in older adults, especially those with clinical vulnerability or living in institutional settings. In contrast, no significant effects were observed for physical activity, and available evidence does not support a consistent trend in favor of IVR. Further research is needed to determine whether specific program designs could enhance its impact in this domain. Pain, perceived effort, and adverse events are included as secondary outcomes, but the evidence is sparse and largely qualitative. Policies and previous research also support the potential of IVR in reducing pain, particularly when using multisensory and emotionally engaging approaches, although the certainty of evidence is low to very low according to GRADE; therefore, these findings should be interpreted with caution. Overall, IVR is well tolerated and shows promise as a safe, adaptable, and motivating tool to support healthy aging, warranting further research in diverse settings.

## Supplementary material

10.2196/80820 Multimedia Appendix 1 Study information.

10.2196/80820 Checklist 1 PRISMA checklist.

## Abbreviations

GRADE: Grades of Recommendation Assessment Development and Evaluation
IVR: immersive virtual reality
NRS: numeric rating scale
PDQ-39: Parkinson Disease Questionnaire-39
PICOS: population, intervention, comparison, outcome, study design
PRISMA: Preferred Reporting Items for Systematic Reviews and Meta-Analyses
RCT: randomized controlled trial
RevMan: Review Manager
SF-12: 12-item Short Form Survey
SMD: standardized mean difference
VAS: visual analog scale
VR: virtual reality
WHO: World Health Organization
WHOQOL-BREF: World Health Organization Quality of Life Scale Brief Version
