# Supplementary material for: Impact of 4 Weeks or More Immersive Virtual Reality on Quality of Life and Physical Activity in Older Adults: Systematic Review and Meta-Analysis
Source: JMIR Aging. 2026 Jan 12;9:e80820. doi: 10.2196/80820 (PMC12795489; doi:10.2196/80820)
Supplement: Multimedia Appendix 1 [file aging-v9-e80820-s001.docx]

## 1. Search strategy

## PUBMED

TITLE/ABSTRACT("Virtual Reality"[MeSH] OR "Virtual reality exposure therapy"[MeSH] OR “Simulated reality” OR “Computer-generated environment” OR “Synthetic environment” OR “Extended reality” OR “Virtual environment” OR “Immersive virtual” OR “Data display”[MeSH] OR “Motion captured” OR “Motion based” OR “Gamified exercise” OR “Head-mounted display” OR HMD)

AND

TITLE/ABSTRACT(Aged OR "Nursing homes" OR "Older adults" OR Senior OR Elderly OR Aging OR Geriatric OR Institutionalized OR "Care home" OR "Long-term care" OR "Assisted living")

AND

(((“Physical Activity” OR Fitness OR “Physical exertion”[MeSH] OR exercise[MeSH] OR “Physical Examination” OR “Walk*” OR “Motor activity” OR “Step*” OR METS OR IPAQ OR accelerometer) OR (Sedentary OR “Physical inactivity” OR Sedentarism OR “Sitting time” OR “Prolonged sitting” OR Stationary OR Motionless)) OR (Pain[MeSH] OR Ache OR Discomfort OR NRS OR “Numerical Rating Scale” OR VAS OR “Visual Analog Scale”) OR (“Physical exertion”[MeSH] OR “Exertion” OR “Fatigue” OR “Intensity perception” OR Effort OR RPE OR Borg) OR (“Quality of life”[MeSH] OR “Health related quality of Life” OR QoL OR HRQoL) OR (“Adverse effects” OR “Adverse events” OR “Side effect*” OR “Cybersickness”))

NOT

(Pediatric OR Paediatric OR Children)

Filters applied: Randomized controlled trial

## WEB OF SCIENCE

Topic ("Virtual Reality" OR "Virtual reality exposure therapy" OR “Simulated reality” OR “Computer-generated environment” OR “Synthetic environment” OR “Extended reality” OR “Virtual environment” OR “Immersive virtual” OR “Data display” OR “Motion captured” OR “Motion based” OR “Gamified exercise” OR “Head-mounted display” OR HMD)

AND

Topic (Aged OR "Nursing homes" OR "Older adults" OR Senior OR Elderly OR Aging OR Geriatric OR Institutionalized OR "Care home" OR "Long-term care" OR "Assisted living")

AND

Topic (((“Physical Activity” OR Fitness OR “Physical exertion” OR exercise OR “Physical Examination” OR “Walk*” OR “Motor activity” OR “Step*” OR METS OR IPAQ OR accelerometer) OR (Sedentary OR “Physical inactivity” OR Sedentarism OR “Sitting time” OR “Prolonged sitting” OR Stationary OR Motionless)) OR (Pain OR Ache OR Discomfort OR NRS OR “Numerical Rating Scale” OR VAS OR “Visual Analog Scale”) OR (“Physical exertion” OR “Exertion” OR “Fatigue” OR “Intensity perception” OR Effort OR RPE OR Borg) OR (“Quality of life” OR “Health related quality of Life” OR QoL OR HRQoL) OR (“Adverse effects” OR “Adverse events” OR “Side effect*” OR “Cybersickness”))

AND

Topic ("Pilot" OR "Randomized controlled trial" OR "Randomised controlled trial" OR "Mixed Methods" OR "Clinical trial")

NOT

Topic (Pediatric OR Paediatric OR Children)

## Filters applied: article

## Scopus

TITLE("Virtual Reality" OR "Virtual reality exposure therapy" OR “Simulated reality” OR “Computer-generated environment” OR “Synthetic environment” OR “Extended reality” OR “Virtual environment” OR “Immersive virtual” OR “Data display” OR “Motion captured” OR “Motion based” OR “Gamified exercise” OR “Head-mounted display” OR HMD)

AND

TITLE-ABS-KEY(Aged OR "Nursing homes" OR "Older adults" OR Senior OR Elderly OR Aging OR Geriatric OR Institutionalized OR "Care home" OR "Long-term care" OR "Assisted living")

AND

TITLE-ABS-KEY(((“Physical Activity” OR Fitness OR “Physical exertion” OR exercise OR “Physical Examination” OR “Walk*” OR “Motor activity” OR “Step*” OR METS OR IPAQ OR accelerometer) OR (Sedentary OR “Physical inactivity” OR Sedentarism OR “Sitting time” OR “Prolonged sitting” OR Stationary OR Motionless)) OR (Pain OR Ache OR Discomfort OR NRS OR “Numerical Rating Scale” OR VAS OR “Visual Analog Scale”) OR (“Physical exertion” OR “Exertion” OR “Fatigue” OR “Intensity perception” OR Effort OR RPE OR Borg) OR (“Quality of life” OR “Health related quality of Life” OR QoL OR HRQoL) OR (“Adverse effects” OR “Adverse events” OR “Side effect*” OR “Cybersickness”))

AND

TITLE-ABS-KEY("Pilot" OR "Randomized controlled trial" OR "Randomised controlled trial" OR "Mixed Methods" OR "Clinical trial")

NOT

TITLE-ABS-KEY(Pediatric OR Paediatric OR Children)

## Filters applied: Article

## PEDro

“Virtual reality” AND Older AND “Physical activity”

“Virtual reality” AND Elderly AND “Physical activity”

“Immersive virtual” AND Older AND “Physical activity”

“Immersive virtual” AND Elderly AND “Physical activity”

“Virtual reality” AND Older AND Pain

“Virtual reality” AND Elderly AND Pain

“Immersive virtual” AND Older AND Pain

“Immersive virtual” AND Elderly AND Pain

“Virtual reality” AND Older AND Effort

“Virtual reality” AND Elderly AND Effort

“Immersive virtual” AND Older AND Effort

“Immersive virtual” AND Elderly AND Effort

“Virtual reality” AND Older AND “Quality of life”

“Virtual reality” AND Elderly AND “Quality of life”

“Immersive virtual” AND Older AND “Quality of life”

“Immersive virtual” AND Elderly AND “Quality of life”

“Virtual reality” AND Older AND “Adverse events”

“Virtual reality” AND Elderly AND “Adverse events”

“Immersive virtual” AND Older AND “Adverse events”

“Immersive virtual” AND Elderly AND “Adverse events”

Filters applied: clinical trial

## Clinicaltrials.gov

Condition/disease: “older adults”

Intervention/treatment: “virtual reality”

Filters applied: with results

## ICTRP

“Virtual reality” AND “Older adults”

Filters applied: with results only

## ProQuest Dissertations & Theses Global

Topic ("Virtual Reality" OR "Virtual reality exposure therapy" OR “Simulated reality” OR “Computer-generated environment” OR “Synthetic environment” OR “Extended reality” OR “Virtual environment” OR “Immersive virtual” OR “Data display” OR “Motion captured” OR “Motion based” OR “Gamified exercise” OR “Head-mounted display” OR HMD)

AND

Topic (Aged OR "Nursing homes" OR "Older adults" OR Senior OR Elderly OR Aging OR Geriatric OR Institutionalized OR "Care home" OR "Long-term care" OR "Assisted living")

AND

Topic (((“Physical Activity” OR Fitness OR “Physical exertion” OR exercise OR “Physical Examination” OR “Walk*” OR “Motor activity” OR “Step*” OR METS OR IPAQ OR accelerometer) OR (Sedentary OR “Physical inactivity” OR Sedentarism OR “Sitting time” OR “Prolonged sitting” OR Stationary OR Motionless)) OR (Pain OR Ache OR Discomfort OR NRS OR “Numerical Rating Scale” OR VAS OR “Visual Analog Scale”) OR (“Physical exertion” OR “Exertion” OR “Fatigue” OR “Intensity perception” OR Effort OR RPE OR Borg) OR (“Quality of life” OR “Health related quality of Life” OR QoL OR HRQoL) OR (“Adverse effects” OR “Adverse events” OR “Side effect*” OR “Cybersickness”))

NOT

Topic (Pediatric OR Paediatric OR Children)

## medRxiv

title "Virtual reality" and abstract or title "Older adults"

## 2. Hardware, software and outcomes of studies.

| Study ID | Hardware VR | Software VR | Outcomes: instrument |
| --- | --- | --- | --- |
| Barsasella et al, 2021 [1] | HTC Vive | The Lab, Everest VR, The Body VR:Journey Inside a Cell, To the Top, Waltz of the Wizard, Google Earth VR, Found, Sparc, Final Soccer VR | Quality of life: EuroQol-5D-3L  Happiness  Functional fitness |
| Campo-Prieto et al, 2022 [2] | HTC Vive Pro | BOX VR | Balance and gait  Functional mobility  Handgrip strength  Quality of life: SF-12 Spanish version  Side effects: SSQ  Usability  Gaming experience  VR sessions |
| Chiu et al, 2023 [3] | NI | NI | Cognitive function  Cognitive impairment  Quality of life: WHOQOL-BREF |
| Drazich et al, 2023 [4] | Oculus Quest 2 | Holofit | Feasibility: SSQ  Physical activity: Yale Physical Activity Survey  Depressive symptoms |
| Kershner et al, 2024 [5] | Meta Quest 2 | NI | Feasibility  Acceptability: VRSQ  Social connection  Physical activity (daily steps, daily minutes of moderate to vigorous physical activity and total physical activity): Garmin Vivosmart 4 watch and self-reported activity diary  Functional capacity |
| Khirallah Abd el Fatah et al, 2024 [6] | Oculus Quest Head Mounted Display | VR Wander | Cognitive function  Psychological well-being  Side effects: CSQ-VR  Participant's experience in immersive VR |
| Kwan et al, 2021 [7] | HTC Vive Focus Plus | NI | Cognitive function  Physical frailt  Walking Speed  Muscle strength  Side effects: VRSQ  Feasibility |
| Kwan et al, 2024 [8] | HTC Vive Focus Plus | NI | Cognitive function  Physical frailty  Walking Speed  Verbal short-term memory  Inhibition of cognitive interference.  Executive function  Side effects: VRSQ and open-ended questions |
| Lo et al, 2024 [9] | VR Shinecon 5.0 | VR software developed by research team | Recruitment rate  Response rate  Dropout rate  Intervention adherence:  Side effects: participants’ exercise records  Pain intensity: NRS and WOMAC pain subscale  Physical function: steps with ActivPAL monitor  Health-related quality of life: EQ-VAS (EuroQol-5D)  Qualitative outcomes |
| Mazurek et al, 2023 [10] | HTC Vive | VRTierOne device | Anxiety and depression  Stress  Optimistic self-beliefs  Daily living activities  Mobility  Gait and balance  Pain: VAS |
| Rodríguez-Fuentes et al, 2024 [11] | Meta Quest II | HoloFit | Dynamic balance  Lower extremity strength  Gait and balance  Quality of life: PDQ-39  Symptomatology and follow-ups  Side effects: SSQ  System usability  Personal impressions |
| Sekar et al, 2024 [12] | Jiodive VR Headset | NI | Balance  Mobility  Quality of life: EQ-5D |
| Stamm et al, 2022 [13] | HTC Vive | ViRST VR | Pain intensity: NRS  Severity of chronic pain  Functional capacities  Fear-avoidance beliefs  Quality of life: SF-12  Immersion of VR |
| Zheng et al, 2025 [14] | NI | NI | Activities daily living  Instrumental activities daily living  Basic activities daily living:  Cognitive function  Depression  Quality of life: SF-12 |

## 3. Detailed deviations from protocol

Funnel plots and Egger’s test were not performed due to the limited number of studies included in each meta-analysis (n < 10), in accordance with Cochrane methodological guidance. Regarding the quality of life outcome, subgroup analyses based on intervention type or follow-up duration could not be conducted due to insufficient data reporting. Similarly, no subgroup analyses were performed for physical activity, as there were fewer than two studies per subgroup.

Only the physical component of the 12-item Short Form Survey (SF-12) was included in the meta-analysis, as it was the most relevant domain in relation to the primarily physical focus of the interventions assessed. Inclusion of both the physical and the mental component would have led to data duplication and violated psychometric principles, since the two components reflect distinct constructs and are not designed to be combined [15]

In the case of Kershner et al., outcome data were reported as medians and interquartile ranges (IQR) [5]. To enable inclusion of this study in the meta-analysis, approximate means and standard deviations were estimated using validated statistical methods for small samples with assumed symmetric distributions [16,17]. The median was taken as the mean, and the standard deviation was calculated by dividing the IQR by 1.35.

Studies that reported only EQ-VAS scores were also included, as the EQ-VAS is a validated component of the EuroQol-5D instrument [18]. While it does not capture the multidimensional structure of the full EuroQol-5D index, it provides a reliable and interpretable measure of self-perceived health status. Given its conceptual alignment with other measures of quality of life used in the review, and to retain a larger number of eligible studies in the quantitative synthesis, EQ-VAS data were deemed appropriate for inclusion using SMD methods.

Finally, although perceived effort, pain intensity, and adverse events were defined as secondary outcomes in the review protocol, none of these could be included in the quantitative synthesis. Specifically, perceived effort was not systematically assessed in any of the included studies using validated tools. Regarding pain intensity, only two studies reported this outcome. In accordance with Cochrane methodological guidance, meta-analysis was not conducted due to the insufficient number of studies (n < 3), which would have rendered pooled estimates statistically unreliable and potentially misleading. Regarding adverse events, although several studies documented symptoms (e.g., cybersickness), data were reported inconsistently, often narratively or as incidence counts without denominators or statistical comparisons. Therefore, these outcomes were described qualitatively but excluded from meta-analytic pooling due to methodological and reporting limitations.

1. Barsasella D, Liu MF, Malwade S, et al. Effects of virtual reality sessions on the quality of life, happiness, and functional fitness among the older people: a randomized controlled trial from Taiwan. Comput Methods Programs Biomed. Mar 2021;200:105892. [doi: 10.1016/j.cmpb.2020.105892]
2. Campo-Prieto P, Cancela-Carral JM, Rodríguez-Fuentes G. Feasibility and effects of an immersive virtual reality exergame program on physical functions in institutionalized older adults: a randomized clinical trial. Sensors (Basel). Sep 6, 2022;22(18):6742. [doi: 10.3390/s22186742]
3. Chiu HM, Hsu MC, Ouyang WC. Effects of incorporating virtual reality training intervention into health care on cognitive function and wellbeing in older adults with cognitive impairment: a randomized controlled trial. Int J Hum Comput Stud. Feb 2023;170:102957. [doi: 10.1016/j.ijhcs.2022.102957]
4. Drazich BF, Anokye D, Zhu S, et al. Motivating older adults through immersive virtual exercise (motive): a randomized pilot study. Geriatr Nurs. 2023;54:229-236. [doi: 10.1016/j.gerinurse.2023.09.019] [Medline: 37844539]
5. Kershner K, Morton D, Robison J, N’dah KW, Fanning J. Assessing the feasibility and acceptability of virtual reality for remote group-mediated physical activity in older adults: pilot randomized controlled trial. JMIR Form Res. Nov 8, 2024;8:e53156. [doi: 10.2196/53156] [Medline: 39514256]
6. Khirallah Abd El Fatah N, Abdelwahab Khedr M, Alshammari M, Mabrouk Abdelaziz Elgarhy S. Effect of immersive virtual reality reminiscence versus traditional reminiscence therapy on cognitive function and psychological well-being among older adults in assisted living facilities: a randomized controlled trial. Geriatr Nurs. 2024;55:191-203. [doi: 10. 1016/j.gerinurse.2023.11.010]
7. Kwan RYC, Liu JYW, Fong KNK, et al. Feasibility and effects of virtual reality motor-cognitive training in communitydwelling older people with cognitive frailty: pilot randomized controlled trial. JMIR Serious Games. Aug 6, 2021;9(3):e28400. [doi: 10.2196/28400]
8. Kwan RYC, Liu J, Sin OSK, et al. Effects of virtual reality motor-cognitive training for older people with cognitive frailty: multicentered randomized controlled trial. J Med Internet Res. Sep 11, 2024;26:e57809. [doi: 10.2196/57809]
9. Lo HHM, Ng M, Fong PYH, et al. Examining the feasibility, acceptability, and preliminary efficacy of an immersive virtual reality-assisted lower limb strength training for knee osteoarthritis: mixed methods pilot randomized controlled trial. JMIR Serious Games. Sep 27, 2024;12(2024):e52563. [doi: 10.2196/52563]
10. Mazurek J, Cieślik B, Wrzeciono A, Gajda R, Szczepańska-Gieracha J. Immersive virtual reality therapy is supportive for orthopedic rehabilitation among the elderly: a randomized controlled trial. J Clin Med. Dec 14, 2023;12(24):7681. [doi: 10.3390/jcm12247681]
11. Rodríguez-Fuentes G, Campo-Prieto P, Cancela-Carral JM. Immersive virtual reality high-intensity aerobic training to slow parkinson’s disease: the ReViPark program. Appl Sci. ;14(11):4708. [doi: 10.3390/app14114708]
12. Sekar M, Suganthirababu P, Subramanian SS, et al. The effectiveness of virtual reality (VR) therapy on balance and mobility in elderly patients: a randomized controlled trial. Fiz Pol. Dec 31, 2024;24(5):191-194. [doi: 10.56984/ 8ZG020C8UWP]
13. Stamm O, Dahms R, Reithinger N, Ruß A, Müller-Werdan U. Virtual reality exergame for supplementing multimodal pain therapy in older adults with chronic back pain: a randomized controlled pilot study. Virtual Real. 2022;26(4):1291-1305. [doi: 10.1007/s10055-022-00629-3]
14. Zheng L, Li X, Xu Y, et al. Effects of virtual reality-based activities of daily living rehabilitation training in older adults with cognitive frailty and activities of daily living impairments: a randomized controlled trial. J Am Med Dir Assoc. Feb 2025;26(2):105397. [doi: 10.1016/j.jamda.2024.105397]
15. Ware JE, Kosinski M, Diane TB. How to Score SF-12 Physical and Mental Health Summary Scales. 2nd ed. The Health Institute, New England Medical Center; 1995.
16. Shi J, Luo D, Weng H, et al. Optimally estimating the sample standard deviation from the five‐number summary. Res Synth Methods. Sep 2020;11(5):641-654. [doi: 10.1002/jrsm.1429]
17. Luo D, Wan X, Liu J, Tong T. Optimally estimating the sample mean from the sample size, median, mid-range, and/or mid-quartile range. Stat Methods Med Res. Jun 2018;27(6):1785-1805. [doi: 10.1177/0962280216669183] [Medline: 27683581]
18. Cheng LJ, Tan RLY, Luo N. Measurement properties of the EQ VAS around the globe: a systematic review and metaregression analysis. Value Health. Aug 2021;24(8):1223-1233. [doi: 10.1016/j.jval.2021.02.003]
